# 1-(3-Chloro­benz­yl)-5-iodo­indoline-2,3-dione

**DOI:** 10.1107/S1600536808034727

**Published:** 2008-10-31

**Authors:** Obaid-ur-Rahman Abid, Ghulam Qadeer, Nasim Hasan Rama, Ales Ruzicka

**Affiliations:** a Department of Chemistry, Quaid-i-Azam University, Islamabad 45320, Pakistan; b Department of General and Inorganic Chemistry, Faculty of Chemical Technology, University of Pardubice, Nam. Cs. Legii’ 565, 53210 Pardubice, Czech Republic

## Abstract

In the title compound, C_15_H_9_ClINO_2_, which possesses anticonvulsant activity, the iodo­indoline ring system is essentially planar (maximum deviation 1.245 Å) and is oriented with respect to the 3-chloro­benzyl ring at a dihedral angle of 76.59 (3)°. In the crystal, there is a π–π contact between iodo­indoline ring systems [centroid–centroid distance = 3.8188 (4) Å].

## Related literature

For general background, see: Hibino & Choshi (2002 ▶); Somei & Yamada (2003 ▶); Popp (1977 ▶); Popp (1984 ▶). For related structures, see: Chakraborty & Talapatra (1985 ▶); Chakraborty *et al.* (1985 ▶); Codding *et al.* (1984 ▶); De (1992 ▶); De & Kitagawa (1991*a* ▶,*b* ▶); Itai *et al.* (1978 ▶). For bond-length data, see: Allen *et al.* (1987 ▶);
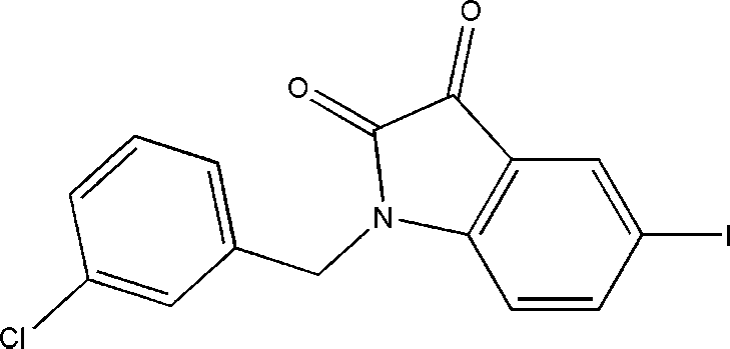


## Experimental

### 

#### Crystal data

C_15_H_9_ClINO_2_
*M* *_r_* = 397.58
Monoclinic, 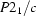

*a* = 8.1241 (6) Å
*b* = 11.7930 (8) Å
*c* = 14.7001 (2) Å
β = 90.751 (3)°
*V* = 1408.23 (14) Å^3^
*Z* = 4
Mo *K*α radiation
μ = 2.46 mm^−1^
*T* = 150 (1) K
0.37 × 0.30 × 0.06 mm

#### Data collection

Bruker–Nonius KappaCCD area-detector diffractometer
Absorption correction: integration (Coppens, 1970 ▶) *T*_min_ = 0.473, *T*_max_ = 0.837
12236 measured reflections
3203 independent reflections
2570 reflections with *I* > 2σ(*I*)
*R*_int_ = 0.051

#### Refinement

*R*[*F* ^2^ > 2σ(*F* ^2^)] = 0.039
*wR*(*F* ^2^) = 0.104
*S* = 1.11
3203 reflections
181 parameters
H-atom parameters constrained
Δρ_max_ = 1.43 e Å^−3^
Δρ_min_ = −0.79 e Å^−3^

### 

Data collection: *COLLECT* (Hooft, 1998 ▶); cell refinement: *COLLECT* and *DENZO* (Otwinowski & Minor, 1997 ▶); data reduction: *COLLECT* and *DENZO*; program(s) used to solve structure: *SIR92* (Altomare *et al.*, 1994 ▶); program(s) used to refine structure: *SHELXL97* (Sheldrick, 2008 ▶); molecular graphics: *PLATON* (Spek, 2003 ▶); software used to prepare material for publication: *SHELXL97*.

## Supplementary Material

Crystal structure: contains datablocks I, global. DOI: 10.1107/S1600536808034727/hk2560sup1.cif

Structure factors: contains datablocks I. DOI: 10.1107/S1600536808034727/hk2560Isup2.hkl

Additional supplementary materials:  crystallographic information; 3D view; checkCIF report

## Figures and Tables

**Fig. 1.**
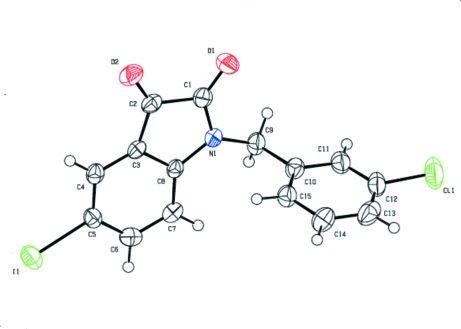
The molecular structure of the title molecule, with the atom-numbering scheme. Displacement ellipsoids drawn at the 50% probability level.

**Fig. 2.**
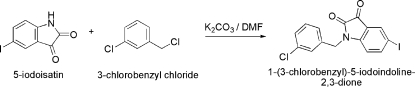
The formation of the title compound.

## supplementary crystallographic information

### Comment

Indolinones are a class of heterocyclic compounds found in many natural products and in a number of marketed drugs (Hibino & Choshi, 2002; Somei & Yamada, 2003). They have diverse chemical structures and complex physiological and pharmacological actions. The search for potential drugs and their mechanism of action has been difficult because of their complexity. These compounds contain both oxoindole and dioxolane moieties which have independently been seen in other anticonvulsants (Popp, 1977, 1984). The title compound, a chloro analogue, was found to be most potent in the MES test. Since no common target site has yet been established, X-ray analysis was undertaken to search structural information which may help in the understanding of the mechanism of action at the molecular level.

In the title compound (Fig. 1), the bond lengths (Allen *et al.*, 1987) and angles are within normal ranges. Rings A (N1/C1-C3/C8), B (C3-C8) and C (C10-C15) are, of course, planar and the dihedral angles between them are A/B = 0.83 (3)°, A/C = 77.05 (3)° and B/C = 76.22 (3)°. The C2-C3 [1.463 (5) Å] bond is slightly shorter but closely similar to the values found in other indoline nuclei (Itai *et al.*,  1978; Chakraborty & Talapatra, 1985; Chakraborty *et al.*, 1985; De & Kitagawa, 1991a,b; De, 1992). The lone pair of electrons on N1 atom is involved in conjugation with the carbonyl group. This is also indicated by the slight lengthening of the C1=O1 [1.208 (5) Å] bond  and the concomitant shortening of the N1-C1 [1.364 (5) Å] and N1-C8 [1.407 (5) Å] single bonds (Codding *et al.*,  1984).

In the crystal structure, the π-π contact between the iodoindoline rings, Cg2—Cg2^i^ [symmetry code: (i) 1 - x, -y, -z, where Cg2 is centroid of the ring B (C3-C8)] may stabilize the structure, with centroid-centroid distance of 3.8188 (4) Å.

### Experimental

A mixture of 5-iodoisatin (1.8 g, 10 mmol) and 3-chlorobenzyl chloride (1.6 g, 10 mmol) was refluxed in DMF (50 ml) in the precense of potassium carbonate for 6 h. DMF was removed from the reaction mixture by distillation. Ice cold water (20 ml) was added and the reaction mixture was extracted with dichloromethane (3 × 20 ml). The extract was dried and evaporated to yield the crude solid, which was recrystallized from methanol (yield; 74%; m.p. 411-412 K).

### Refinement

H atoms were positioned geometrically, with C-H = 0.93 and 0.97 Å for aromatic and methylene H, respectively, and constrained to ride on their parent atoms with U_iso_(H) = 1.2U_eq_(C).

### Crystal data

**Table:**

C_15_H_9_ClINO_2_	*F*(000) = 768
*M**_r_* = 397.58	*D*_x_ = 1.875 Mg m^−^^3^
Monoclinic, *P*2_1_/*c*	Melting point: 411(1) K
Hall symbol: -P 2ybc	Mo *K*α radiation, λ = 0.71073 Å
*a* = 8.1241 (6) Å	Cell parameters from 12323 reflections
*b* = 11.7930 (8) Å	θ = 1–27.5°
*c* = 14.7001 (2) Å	µ = 2.46 mm^−^^1^
β = 90.751 (3)°	*T* = 150 K
*V* = 1408.23 (14) Å^3^	Plate, colorless
*Z* = 4	0.37 × 0.30 × 0.06 mm

### Data collection

**Table:**

Bruker–Nonius KappaCCD area-detector diffractometer	3203 independent reflections
Radiation source: fine-focus sealed tube	2570 reflections with *I* > 2σ(*I*)
graphite	*R*_int_ = 0.051
Detector resolution: 9.091 pixels mm^-1^	θ_max_ = 27.5°, θ_min_ = 2.2°
φ and ω scans	*h* = −10→9
Absorption correction: integration (Coppens, 1970)	*k* = −15→14
*T*_min_ = 0.473, *T*_max_ = 0.837	*l* = −17→19
12236 measured reflections	

### Refinement

**Table:**

Refinement on *F*^2^	Primary atom site location: structure-invariant direct methods
Least-squares matrix: full	Secondary atom site location: difference Fourier map
*R*[*F*^2^ > 2σ(*F*^2^)] = 0.039	Hydrogen site location: inferred from neighbouring sites
*wR*(*F*^2^) = 0.104	H-atom parameters constrained
*S* = 1.11	*w* = 1/[σ^2^(*F*_o_^2^) + (0.0373*P*)^2^ + 3.1264*P*] where *P* = (*F*_o_^2^ + 2*F*_c_^2^)/3
3203 reflections	(Δ/σ)_max_ < 0.001
181 parameters	Δρ_max_ = 1.43 e Å^−^^3^
0 restraints	Δρ_min_ = −0.79 e Å^−^^3^

### Special details

**Table:**

Geometry. All e.s.d.'s (except the e.s.d. in the dihedral angle between two l.s. planes) are estimated using the full covariance matrix. The cell e.s.d.'s are taken into account individually in the estimation of e.s.d.'s in distances, angles and torsion angles; correlations between e.s.d.'s in cell parameters are only used when they are defined by crystal symmetry. An approximate (isotropic) treatment of cell e.s.d.'s is used for estimating e.s.d.'s involving l.s. planes.
Refinement. Refinement of *F*^2^ against ALL reflections. The weighted *R*-factor *wR* and goodness of fit *S* are based on *F*^2^, conventional *R*-factors *R* are based on *F*, with *F* set to zero for negative *F*^2^. The threshold expression of *F*^2^ > σ(*F*^2^) is used only for calculating *R*-factors(gt) *etc*. and is not relevant to the choice of reflections for refinement. *R*-factors based on *F*^2^ are statistically about twice as large as those based on *F*, and *R*- factors based on ALL data will be even larger.

### Fractional atomic coordinates and isotropic or equivalent isotropic displacement parameters (Å^2^)

**Table:**

	*x*	*y*	*z*	*U*_iso_*/*U*_eq_	
I1	0.21126 (4)	−0.00688 (3)	−0.168370 (19)	0.04622 (13)	
Cl1	0.0801 (2)	0.12211 (13)	0.61991 (8)	0.0713 (5)	
N1	0.3994 (4)	0.1400 (3)	0.2264 (2)	0.0304 (7)	
O1	0.5622 (4)	0.2894 (3)	0.2730 (2)	0.0454 (8)	
O2	0.5749 (4)	0.3308 (3)	0.0755 (2)	0.0472 (8)	
C1	0.4945 (5)	0.2340 (3)	0.2144 (3)	0.0331 (8)	
C2	0.5037 (5)	0.2528 (3)	0.1103 (3)	0.0330 (8)	
C3	0.4080 (5)	0.1602 (3)	0.0698 (3)	0.0286 (8)	
C4	0.3728 (5)	0.1328 (3)	−0.0195 (3)	0.0305 (8)	
H4	0.4138	0.1759	−0.0670	0.037*	
C5	0.2736 (5)	0.0393 (3)	−0.0354 (3)	0.0311 (8)	
C6	0.2127 (5)	−0.0241 (3)	0.0360 (3)	0.0360 (9)	
H6	0.1452	−0.0860	0.0235	0.043*	
C7	0.2493 (5)	0.0025 (3)	0.1261 (3)	0.0333 (8)	
H7	0.2090	−0.0406	0.1739	0.040*	
C8	0.3475 (4)	0.0955 (3)	0.1419 (2)	0.0261 (7)	
C9	0.3778 (5)	0.0850 (4)	0.3135 (3)	0.0354 (9)	
H9A	0.4725	0.1016	0.3521	0.043*	
H9B	0.3743	0.0036	0.3042	0.043*	
C10	0.2237 (5)	0.1204 (3)	0.3623 (3)	0.0339 (8)	
C11	0.2173 (6)	0.1038 (4)	0.4557 (3)	0.0385 (9)	
H11	0.3057	0.0708	0.4866	0.046*	
C12	0.0790 (7)	0.1355 (4)	0.5020 (3)	0.0434 (11)	
C13	−0.0563 (7)	0.1805 (4)	0.4584 (4)	0.0528 (13)	
H13	−0.1491	0.2010	0.4910	0.063*	
C14	−0.0506 (6)	0.1953 (4)	0.3650 (3)	0.0449 (11)	
H14	−0.1422	0.2232	0.3337	0.054*	
C15	0.0905 (5)	0.1688 (3)	0.3183 (3)	0.0363 (9)	
H15	0.0956	0.1836	0.2563	0.044*	

### Atomic displacement parameters (Å^2^)

**Table:**

	*U*^11^	*U*^22^	*U*^33^	*U*^12^	*U*^13^	*U*^23^
I1	0.03707 (18)	0.0640 (2)	0.03741 (18)	0.01182 (13)	−0.00630 (12)	−0.01648 (13)
Cl1	0.1128 (13)	0.0684 (9)	0.0331 (6)	−0.0229 (8)	0.0206 (7)	−0.0073 (6)
N1	0.0271 (17)	0.0358 (17)	0.0284 (16)	−0.0010 (13)	0.0047 (12)	−0.0009 (13)
O1	0.0425 (18)	0.0493 (18)	0.0443 (17)	−0.0059 (14)	−0.0025 (14)	−0.0135 (14)
O2	0.0465 (19)	0.0435 (17)	0.0519 (19)	−0.0149 (14)	0.0100 (15)	0.0029 (14)
C1	0.029 (2)	0.035 (2)	0.0356 (19)	0.0031 (16)	0.0049 (15)	−0.0059 (16)
C2	0.027 (2)	0.0309 (19)	0.041 (2)	−0.0003 (15)	0.0081 (16)	−0.0015 (16)
C3	0.0227 (18)	0.0307 (18)	0.0325 (19)	0.0017 (14)	0.0062 (14)	0.0007 (15)
C4	0.027 (2)	0.0349 (19)	0.0302 (18)	0.0065 (15)	0.0041 (15)	−0.0003 (15)
C5	0.027 (2)	0.0361 (19)	0.0299 (18)	0.0080 (16)	0.0000 (15)	−0.0064 (16)
C6	0.030 (2)	0.033 (2)	0.045 (2)	−0.0015 (16)	−0.0017 (17)	−0.0033 (17)
C7	0.029 (2)	0.0333 (19)	0.038 (2)	−0.0004 (16)	0.0059 (16)	0.0052 (16)
C8	0.0222 (18)	0.0304 (17)	0.0260 (16)	0.0047 (14)	0.0038 (13)	0.0000 (14)
C9	0.034 (2)	0.045 (2)	0.0275 (18)	0.0062 (18)	0.0030 (16)	0.0041 (17)
C10	0.038 (2)	0.0315 (19)	0.0318 (19)	−0.0012 (16)	0.0048 (16)	0.0026 (15)
C11	0.048 (3)	0.037 (2)	0.031 (2)	−0.0049 (19)	0.0024 (18)	−0.0002 (17)
C12	0.065 (3)	0.037 (2)	0.029 (2)	−0.013 (2)	0.0120 (19)	−0.0039 (17)
C13	0.054 (3)	0.042 (2)	0.063 (3)	−0.009 (2)	0.026 (2)	−0.013 (2)
C14	0.038 (2)	0.046 (2)	0.051 (3)	0.0084 (19)	0.009 (2)	0.002 (2)
C15	0.038 (2)	0.040 (2)	0.0306 (19)	0.0019 (18)	0.0019 (16)	0.0027 (17)

### Geometric parameters (Å, °)

**Table:**

I1—C5	2.086 (4)	C7—C8	1.374 (5)
Cl1—C12	1.740 (4)	C7—H7	0.9301
N1—C1	1.364 (5)	C9—C10	1.510 (6)
N1—C8	1.407 (5)	C9—H9A	0.9700
N1—C9	1.448 (5)	C9—H9B	0.9701
O1—C1	1.208 (5)	C11—C10	1.388 (6)
O2—C2	1.204 (5)	C11—H11	0.9300
C1—C2	1.550 (6)	C12—C13	1.372 (8)
C2—C3	1.463 (5)	C12—C11	1.373 (7)
C3—C8	1.401 (5)	C13—C14	1.386 (7)
C4—C3	1.378 (5)	C13—H13	0.9299
C4—H4	0.9299	C14—H14	0.9300
C5—C4	1.384 (6)	C15—C10	1.377 (6)
C5—C6	1.384 (6)	C15—C14	1.379 (6)
C6—H6	0.9300	C15—H15	0.9299
C7—C6	1.390 (6)		
			
C1—N1—C8	110.7 (3)	C3—C8—N1	111.1 (3)
C1—N1—C9	123.6 (3)	N1—C9—C10	114.0 (3)
C8—N1—C9	125.1 (3)	N1—C9—H9A	108.8
O1—C1—N1	126.9 (4)	C10—C9—H9A	108.8
O1—C1—C2	126.8 (4)	N1—C9—H9B	108.8
N1—C1—C2	106.2 (3)	C10—C9—H9B	108.5
O2—C2—C3	130.8 (4)	H9A—C9—H9B	107.6
O2—C2—C1	124.0 (4)	C15—C10—C11	118.9 (4)
C3—C2—C1	105.2 (3)	C15—C10—C9	122.9 (4)
C4—C3—C8	121.5 (4)	C11—C10—C9	118.2 (4)
C4—C3—C2	131.7 (4)	C12—C11—C10	119.5 (4)
C8—C3—C2	106.8 (3)	C12—C11—H11	120.2
C3—C4—C5	117.4 (4)	C10—C11—H11	120.3
C3—C4—H4	121.1	C13—C12—C11	122.0 (4)
C5—C4—H4	121.5	C13—C12—Cl1	119.6 (4)
C4—C5—C6	121.0 (4)	C11—C12—Cl1	118.4 (4)
C4—C5—I1	120.0 (3)	C12—C13—C14	118.3 (4)
C6—C5—I1	119.0 (3)	C12—C13—H13	120.7
C5—C6—C7	121.7 (4)	C14—C13—H13	121.0
C5—C6—H6	119.3	C15—C14—C13	120.2 (5)
C7—C6—H6	119.0	C15—C14—H14	119.9
C8—C7—C6	117.3 (4)	C13—C14—H14	119.9
C8—C7—H7	121.2	C10—C15—C14	120.9 (4)
C6—C7—H7	121.5	C10—C15—H15	119.5
C7—C8—C3	121.0 (3)	C14—C15—H15	119.6
C7—C8—N1	127.9 (3)		
